# Enamel Matrix Derivative in Dental Pulp Regeneration: Biological Basis, Mechanistic Insights, and Translational Considerations

**DOI:** 10.1155/sci/5777572

**Published:** 2026-06-08

**Authors:** LinLin Zhang, KaiLiang Tang, XinYu Zhao, Yi Du

**Affiliations:** ^1^ Department of Endodontics, Central Laboratory, Jinan Stomatological Hospital, Jinan, Shandong, China, jnskqyy.com; ^2^ Department of Stomatology, Jinan Hospital, Jinan, Shandong, China

**Keywords:** biomaterials, dental pulp regeneration, dental pulp stem cells, enamel matrix derivative, regenerative endodontics

## Abstract

Enamel matrix derivative (EMD) has attracted increasing attention in regenerative endodontics because of its potential to influence multiple biological processes involved in dental pulp healing. Derived from enamel matrix proteins (EMPs), EMD has been associated with the regulation of stem/progenitor cell migration, odontogenic differentiation, angiogenic activity, and inflammatory responses. This review summarizes current evidence regarding the role of EMD in dental pulp regeneration, with emphasis on its biological characteristics, underlying mechanisms, and integration with biomaterial systems. Available studies suggest that EMD may contribute to a regeneration‐permissive microenvironment by modulating cellular behavior and extracellular matrix‐related processes, while biomaterial‐based delivery strategies have been explored to improve its stability and local bioactivity. However, most existing evidence is derived from in vitro studies or nonpulp regenerative models, and direct evidence supporting functional pulp regeneration remains limited. In addition, EMD, Emdogain, recombinant amelogenin (Am), and Am‐derived peptides are closely related but should not be considered interchangeable interventions. Taken together, EMD represents a biologically relevant but not yet fully validated approach in regenerative endodontics, and further studies are required to clarify its mechanisms under clinically relevant conditions and to distinguish true pulp regeneration from partial reparative outcomes.

## 1. Introduction

Dental pulp is essential for maintaining the physiological functions and sensory perception of the teeth. Traditionally, irreversible pulpitis or pulp necrosis caused by infection or trauma has been treated with root canal therapy (RCT). While RCT effectively eliminates infection and relieves pain, it involves the removal of the pulp tissue, leading to a loss of tooth vitality, increased fragility, and a higher risk of reinfection [1]. As a result, the restoration of vascularization, innervation, and immune competence within the pulp tissue has become a major objective in regenerative endodontic research.

Regenerative endodontic therapy (RET), based on the principles of tissue engineering, aims to regenerate the pulp–dentin complex and support continued root development, offering a biologically based alternative to traditional RCT [2]. Among various bioactive molecules, the enamel matrix derivative (EMD) has attracted considerable attention. EMD is derived from enamel matrix proteins (EMPs), which are extracted from porcine tooth germs at approximately 6 months of gestation. These EMPs, secreted by the Hertwig’s epithelial root sheath, are important epithelial signaling molecules during root development and have been implicated in odontogenic differentiation within the dental microenvironment [3]. They facilitate the differentiation of adjacent mesenchymal cells into odontoblasts and cementoblasts, which are essential for the root formation and periodontal tissue regeneration.

Since the introduction of the commercial product Emdogain (a combination of EMD and propylene glycol alginate) in the late 1990s, numerous studies have investigated its efficacy in promoting periodontal regeneration [4]. EMD facilitates the regeneration of the cementum, periodontal ligament, and alveolar bone by regulating the expression of various growth factors [5] and inflammatory mediators [6], targeting cells involved in periodontal repair. EMD exhibits multiple biological functions, including promoting angiogenesis, providing anti‐inflammatory and antibacterial effects, and accelerating tissue healing [7]. Given the similarities in the embryonic origin and regenerative regulation of periodontal and dental pulp tissues, which rely on epithelial–mesenchymal interactions and growth factor signaling, it is reasonable to consider that EMD’s biological activity may extend beyond periodontal tissues, although the extent of this effect in pulp‐specific contexts remains to be clarified [8]. It may also regulate stem cell behavior and enhance vascular and nerve regeneration via similar signaling pathways in the dental pulp microenvironment. Increasing experimental and animal studies support the positive effects of EMD on odontoblast induction, angiogenesis, immune regulation, and integration with biomaterials. However, the currently available evidence remains heterogeneous, and much of it is derived from preclinical or nonpulp models. Therefore, EMD is better interpreted as a biologically relevant candidate rather than a definitively established regulator of pulp regeneration. This review aims to critically examine the role of EMD in dental pulp regeneration, with a focus on its biological characteristics, molecular mechanisms, strategies for combining with biomaterials, and its potential for clinical translation. Ongoing debates include whether EMD acts primarily through direct cell signaling versus matrix remodeling and whether its effects are pulp‐specific or reflect general regenerative properties shared with periodontal tissues.

## 2. Methods

Because this manuscript was designed as a narrative review, a structured literature survey was performed rather than a formal systematic review methodology. Relevant studies were identified through commonly used scientific databases, including PubMed, Web of Science, and Scopus, with a focus on literature related to EMD and dental pulp regeneration.

Search terms included combinations of “enamel matrix derivative,” “Emdogain,” “amelogenin,” “amelogenin‐derived peptide,” “recombinant amelogenin,” “dental pulp regeneration,” “regenerative endodontics,” “odontogenic differentiation,” “angiogenesis,” “neurogenesis,” “immunomodulation,” and “biomaterials.” These terms were used flexibly to capture a broad range of relevant studies rather than to define a strictly bounded search strategy.

Preference was given to studies published in English and judged to be relevant to the biological or translational roles of EMD‐related interventions in dental pulp regeneration. The retrieved literature included in vitro studies, animal studies, preclinical research, narrative reviews, and limited clinically relevant reports.

During article selection and synthesis, the evidence was interpreted in relation to its relevance to dental pulp regeneration. Accordingly, studies were considered within two broad categories:

1. Direct evidence referring to studies involving pulp cells, pulp tissue, root canal regeneration, or other models directly relevant to dental pulp regeneration;
2. Indirect supportive evidence referring to studies in related tissues or regenerative contexts (e.g., periodontal regeneration, bone repair, enamel remineralization, and wound healing) that may provide mechanistic or biomaterial‐related insights but do not directly demonstrate pulp regeneration.

Studies that focused exclusively on unrelated regenerative contexts without plausible relevance to pulp regeneration are not emphasized in the main discussion. Given the heterogeneity of the available literature, the aim of this review was not quantitative synthesis but rather a critical and interpretive overview of the current evidence and its potential translational implications.

## 3. Biological Characteristics of EMD

It should be noted that EMD, emdogain, recombinant amelogenin (Am), and Am‐derived peptides represent related but not equivalent interventions, and their biological effects may not be directly interchangeable.

The components of EMD are complex and consist predominantly of Ams, together with smaller amounts of nonamelogenin proteins such as enamelin, ameloblastin, and proteases, as well as growth factor‐related biological activities reported in association with TGF‐β1 and BMP‐2 [9]. Am, a hydrophobic extracellular matrix protein secreted by ameloblasts, is the most abundant component of the EMD, comprising ~90% of its total mass [10].

Am has been reported to stimulate target cells to secrete multipotent growth factors and cytokines, partially reflecting epithelial–mesenchymal interactions observed during tooth development [11] but also providing signals that regulate gene expression. It is involved in modulating cell signaling pathways and promoting tissue regeneration [12]. Furthermore, Am contributes to the deposition and mineralization [13] of both enamel and dentin matrices.

In addition to Am, nonamelogenin proteins, such as enamelin and ameloblastin, although constituting less than 10% of the EMD mass, are considered to participate in cell signaling and mineralization. Collectively, EMD may function as a biologically active protein system that modulates cell signaling and as an extracellular matrix‐associated component, providing a potential biological basis for dental pulp tissue regeneration. However, the relative contribution of individual components within the EMD remains to be fully clarified.

## 4. Molecular Mechanism of EMD in Promoting Pulp Regeneration

EMD is a complex bioactive system comprising various protein components, which exhibit growth factor‐like biological activities. Villa et al. [14] demonstrated that different EMD components exhibit specific biological functions. The high‐molecular‐weight fraction (>20 kDa) has been reported to induce the secretion of vascular endothelial growth factor (VEGF) and interleukin‐6 (IL‐6), thereby contributing to angiogenic responses. In contrast, low‐molecular‐weight components have been associated with the production of interleukin‐8 (IL‐8) and monocyte chemoattractant protein‐1 (MCP‐1), which are involved in leukocyte recruitment and tissue repair.

Additionally, EMD has been reported to be associated with TGF‐β‐ and BMP‐related signaling pathways, which are involved in regulating key cellular processes. These growth factors, including BMP, VEGF, and fibroblast growth factor (FGF) [15], are thought to regulate key processes, including cell migration, proliferation, angiogenesis, and odontogenic differentiation. Specifically, TGF‐β1 and FGF have been implicated in cell cycle regulation [16]. VEGF is associated with angiogenic responses [17], while BMPs and FGFs contribute to dentinogenesis‐related signaling [18].

These mechanisms are discussed in greater detail in the following sections.

### 4.1. EMD Promotes the Migration and Proliferation of Dental Stem Cells

The migration and proliferation of stem cells are essential for dental pulp regeneration. EMD is thought to contribute to the formation of a favorable microenvironment for stem cell regeneration by modulating the expression of various signaling molecules. Notably, TGF‐β1, a key component of EMD, has been extensively studied and is considered an important regulator in this context.

TGF‐β1 regulates the cell cycle and gene transcription by activating the ERK1/2 and AKT signaling pathways. It has been reported to promote the proliferation and migration of dental pulp stem cells (DPSCs) and stem cells from the apical papilla (SCAP) [19]. Ding et al. [20] demonstrated that TGF‐β1 enhances SCAP proliferation and significantly increases alkaline phosphatase (ALP) activity. It also upregulates the expression of type I collagen (COL1), osteocalcin (OCN), and dentin sialophosphoprotein (DSPP) [20]. Additionally, Bai et al. [16] found that the effects of TGF‐β1 on DPSCs were dose‐dependent: low doses (≤1 ng/mL) promoted cell viability and early differentiation, while higher doses (≥5 ng/mL) inhibited proliferation by inducing cell cycle arrest.

Furthermore, FGF serves as a vital paracrine signaling molecule that is involved in regulating cell proliferation and the maintenance of stemness. EMD enhances FGF expression and activates the MAPK pathway, thereby boosting the proliferation and metabolic activity of DPSCs [21]. Khoshbin et al. [22] revealed that combining CEM cement with EMD significantly improved the proliferation and differentiation of SCAP, particularly by increasing the expression of odontogenic/osteogenic genes, such as DSPP, Dentin Matrix Protein 1 (DMP1), Bone Sialoprotein (BSP), and ALP activity.

However, it should be noted that most of these observations are derived from in vitro or preclinical models, and their direct relevance to functional pulp regeneration remains to be clarified.

Collectively, these mechanisms may provide a cellular basis for dental pulp regeneration.

### 4.2. EMD‐Induced Odontoblast Differentiation and Mineralization

A primary goal of dental pulp regeneration is to direct stem cells to differentiate into odontoblast‐like cells. TGF‐β1, a key active component of EMD, has been reported to promote odontoblast differentiation in DPSCs by regulating the cell cycle and gene expression. This differentiation process is further enhanced through the synergistic effects of TGF‐β1 and FGF‐2, suggesting that multiple growth factors in EMD may coordinate cell differentiation through an integrated signaling network [17].

EMD has been shown to enhance DPSCs’ activity, increase ALP levels early in culture, and promote their differentiation into odontoblast‐like cells [23]. EMD has been reported to activate multiple signaling pathways, including MAPK [24], ERK, and TGF‐β/Smad pathways [25], which leads to increased ALP activity, as well as upregulation of DSPP, osterix (OSX), and runt‐related transcription factor 2 (RUNX2), in addition to the formation of new hard tissue, thereby enhancing osteogenic and odontoblastic differentiation capacity [26].

Moreover, EMD can promote osteogenic differentiation and enhance the mineralization potential of bone marrow mesenchymal stem cells (BMSCs) by activating the Wnt/β‐catenin pathway [27]. Experimental results suggest that adding EMD to the osteogenic induction medium for 14 days significantly enhances DPSCs’ mineralization ability and upregulates the expression of ALP, DSPP, BMP1, osteopontin (OPN), and transcription factors such as OSX and RUNX2 [28]. Further studies confirmed that EMD promotes mineralized nodule formation and increases OPN and DSPP expression in DPSCs under osteogenic induction conditions [29].

However, it remains unclear whether these molecular and cellular changes consistently translate into fully functional dentin–pulp complex regeneration under in vivo conditions.

In conclusion, EMD appears to exert multiple biological effects at the cellular level, including promoting proliferation, inducing differentiation, and enhancing mineralization potential. These effects are mediated through the coordinated regulation of several signaling pathways, providing a robust biological foundation for its application in dental pulp regeneration. Mechanistically, activation of Wnt/β‐catenin signaling may involve nuclear translocation of β‐catenin and subsequent regulation of downstream target genes associated with odontogenic differentiation.

### 4.3. EMD Promotes Angiogenesis and Nerve Regeneration

Successful pulp regeneration requires not only stem cell differentiation and hard tissue formation but also revascularization and reinnervation. Blood vessels supply essential nutrients and oxygen while nerves restore the sensory and defensive functions of the tooth. The simultaneous regeneration of both is critical for restoring the physiological functions of the dental pulp. An increasing body of evidence has suggested the significant role of EMD in promoting both angiogenesis and nerve regeneration.

Regarding angiogenesis, in vitro studies have shown that EMD exerts a strong chemotactic effect on human umbilical vein endothelial cells (HUVECs) [30], significantly promoting their proliferation, upregulating VEGF expression [31], and enhancing angiogenic capacity [32]. At the molecular level, EMD binds to glucose‐regulated protein 78 (GRP78), which enhances the phosphorylation of hypoxia‐inducible factor‐1α (HIF‐1α) and protein kinase A (PKA), thereby upregulating angiogenesis‐related factors such as IL‐8, MCP‐1, and IL‐6. These actions promote endothelial cell migration and neovascularization [33]. Animal studies have further confirmed that EMD stimulates collagen fiber formation in connective tissues, promotes angiogenesis, and accelerates oral mucosal wound healing [34]. Collectively, these findings indicate that EMD may support angiogenic processes under experimental conditions, although the extent to which these effects contribute to pulp‐specific vascular regeneration remains to be clarified.

In terms of nerve regeneration, animal experiments have detected neural differentiation markers, such as SRY‐Box Transcription Factor 2 (SOX2), peripherin, calcitonin gene‐related peptide (CGRP), and glial fibrillary acidic protein (GFAP), in regenerated pulp tissue following EMD treatment [35]. Further studies showed that neural stem cell‐related immune responses were observed 1 month after root canal treatment with recombinant EMP (rM180), and vascularized pulp‐like tissue formed after 3 months [36]. Mechanistic studies have indicated that EMD may promote pulp revascularization and nerve regeneration through activation of the Wnt/β‐catenin signaling pathway, guiding stem cell differentiation into the dental pulp or odontoblast lineage [37]. However, most of these observations are derived from nonpulp or animal models, and their translational relevance requires further investigation.

This “vascular–nerve regeneration” concept may contribute to the restoration of key biological functions of the dental pulp under experimental conditions and provides a potentially useful perspective for understanding regenerative pulp therapy (RET).

### 4.4. Regulation of Immunity and Anti‐Inflammatory Effects

The inflammatory microenvironment within the dental pulp presents a significant challenge for regenerative therapy. Immune responses triggered by infection or injury can impair the regenerative potential of stem cells, leading to tissue necrosis. Consequently, maintaining or restoring stem cell viability in an inflammatory environment is a critical challenge. Recent studies have emphasized the immunomodulatory and anti‐inflammatory properties of EMD.

Regarding immune regulation, EMD inhibits pro‐inflammatory responses via multiple pathways. EMD has been reported to activate the TGF‐β signaling pathway, significantly suppressing lipopolysaccharide (LPS)‐induced IL‐1β expression in RAW264.7 macrophages and primary macrophages, as well as tumor necrosis factor‐α (TNF‐α) expression [38]. Additionally, EMD reduces the secretion of inflammatory chemokines by oral epithelial cells [39]. Other studies have confirmed that EMD significantly reduces the levels of inflammatory cytokines such as TNF‐α, IL‐1β, and IL‐6 in LPS‐stimulated models [6], further supporting its role in modulating inflammatory responses. Research by Sordi et al. [40] showed that EMD not only reduced the levels of these inflammatory factors but also decreased the expression of pyroptosis‐related genes, including NOD‐like receptor family, pyrin domain‐containing 3 (NLRP3), Caspase recruitment domain family member 1 (CAS1), and Interleukin‐18 (IL‐18). This significantly attenuates LPS‐induced inflammatory responses [40]. These findings suggest that EMD exhibits potent anti‐inflammatory effects, particularly in regulating immune responses and facilitating tissue repair.

In immune cell remodeling, EMD induces the polarization of macrophages from a pro‐inflammatory M1 phenotype to an anti‐inflammatory M2 phenotype. EMD significantly increases the expression of the anti‐inflammatory cytokine interleukin‐10 (IL‐10) and promotes tissue repair and wound healing [41]. Furthermore, EMD upregulates anti‐inflammatory genes such as tumor necrosis factor Alpha protein 6 (TNFAIP6) and Superoxide Dismutase 2 (SOD2), as well as the production of the anti‐inflammatory mediator prostaglandin E2 (PGE2). Through the PGE2/cAMP signaling pathway, EMD promotes M2 macrophage polarization, accelerating inflammation resolution, and tissue regeneration [42]. Additionally, Yotsumoto et al. [43] demonstrated that EMD significantly reduced T‐cell activation by inhibiting the expression of MHC II molecules on macrophage surfaces, thereby decreasing the immune response.

Some studies suggest that EMD may transiently enhance local inflammation during the early stages [43], acting as a potential “priming” mechanism that promotes the timely resolution of inflammation and wound healing. This is achieved by rapidly recruiting immune cells to clear necrotic tissue and pathogens. This biphasic regulatory effect highlights the complexity of EMD‐mediated immunomodulation, and its effects are likely to depend on the specific microenvironment and stage of inflammation.

In general, EMD appears to regulate the pulpal inflammatory microenvironment by modulating the production of inflammatory factors, inducing immune cell phenotypic switching, and restoring the inflammatory balance. This regulation of immune homeostasis not only mitigates inflammatory injury but also creates a favorable regenerative microenvironment that supports stem cell proliferation, angiogenesis, and nerve regeneration. These actions represent key mechanisms through which EMD promotes pulp regeneration.

In conclusion, the multiple biological effects of EMD described in this chapter are unlikely to function in isolation but may interact as part of a coordinated regulatory network. Based on the available evidence, a hypothetical interaction model can be proposed in which TGF‐β/Smad signaling is involved in odontoblast differentiation, potentially acting in concert with BMP signaling. The FGF/MAPK pathway appears to contribute to cell proliferation and metabolic activity, thereby supporting the cellular basis for regeneration. The Wnt/β‐catenin pathway has been suggested to participate in regulating stem cell fate decisions, although its precise role may vary depending on the cellular context.

These cellular processes are further supported by angiogenesis mediated by VEGF and related factors, which provide metabolic and structural support. Furthermore, the regenerative microenvironment associated with EMD may influence immune responses, including the promotion of M2 macrophage polarization.

Taken together, these observations provide a conceptual framework for understanding the potential role of EMD in pulp regeneration; however, the relative contribution and interaction of these pathways remain to be fully elucidated.

**Figure 1:**
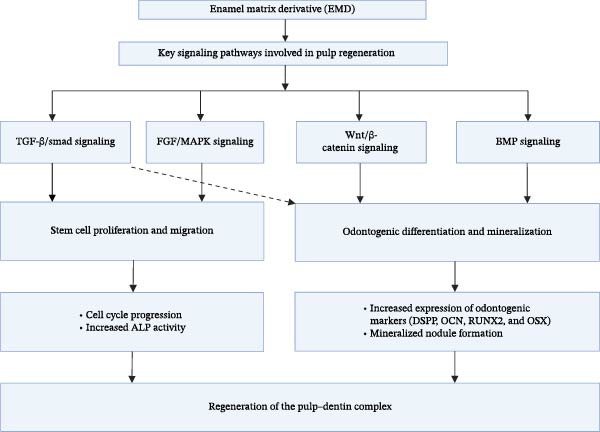
Signaling pathways involved in enamel matrix derivative (EMD)‐mediated dental pulp regeneration. EMD has been reported to modulate multiple signaling pathways, including TGF‐β/Smad, FGF/MAPK, Wnt/β‐catenin, and BMP signaling, which are associated with key cellular processes during pulp regeneration. These pathways contribute to stem cell proliferation and migration, as well as odontogenic differentiation and mineralization, as indicated by increased expression of markers such as DSPP, OCN, RUNX2, and OSX and the formation of mineralized nodules. Ultimately, these coordinated biological processes contribute to the regeneration of the pulp–dentin complex. Solid arrows indicate major reported regulatory relationships, whereas dashed arrows indicate potential or context‐dependent interactions requiring further validation.

**Figure 2:**
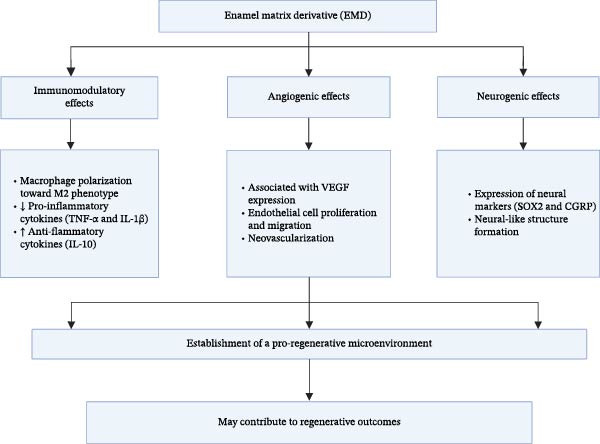
Biological effects of enamel matrix derivative (EMD) on the regenerative microenvironment. EMD is associated with immunomodulatory, angiogenic, and neurogenic effects, including macrophage polarization toward an anti‐inflammatory phenotype, modulation of cytokine expression, endothelial cell proliferation and migration, and the expression of neural‐related markers. These processes collectively contribute to the establishment of a pro‐regenerative microenvironment. Such coordinated effects may contribute to regenerative outcomes in dental pulp tissues. VEGF, vascular endothelial growth factor; SOX2, SRY‐box transcription factor 2; CGRP, calcitonin gene‐related peptide.

A schematic overview of the proposed signaling pathways and microenvironmental effects of EMD is presented in Figures 1 and 2.

## 5. Application Prospects of EMD Combined With Biomaterials

Combining EMD with different types of biomaterials has become a key strategy to enhance dental pulp regeneration, aiming to solve three clinical challenges providing an effective 3D scaffold structure, achieving controlled release of bioactive factors, and actively optimizing the local regeneration microenvironment. EMD alone may not fully meet the requirements of RET for scaffold support, continuous release, and microenvironment regulation. Accordingly, EMD–biomaterial composites have emerged as an important direction in tissue engineering, with the goal of improving the stability and delivery efficiency of EMD and enhancing its capacity to regulate cell behavior and tissue regeneration. It should be noted, however, that most current studies are based on experimental or nonpulp models, and their direct relevance to dental pulp regeneration may therefore vary.

In terms of scaffold materials, researchers try to compound EMD with inorganic or natural polymer materials to obtain composite systems with both mechanical support and biological activity. Liao et al. [44] found that the combination of calcium phosphate/chitosan composite scaffolds with recombinant human amelogenin (rhAM) improved the release stability of EMD and formed a sustained release effect, thereby promoting oral tissue healing and inhibiting the growth of pathogenic microorganisms. Other studies have shown that the combination of EMD and calcium silicate materials can significantly enhance the mineralization effect, suggesting a synergistic effect between EMD and the mineralization microenvironment [45]. This composite system can provide structural support, activate cell differentiation‐ and mineralization‐related signaling pathways, and promote dentin‐like tissue formation. It should be noted that these findings are primarily derived from nonpulp models.

In terms of drug delivery systems, Ho et al.’s electrospinning functional gradient membrane (FGM) combining doxycycline (DOX) and EMD has shown significant antibacterial and osteogenic effects in alveolar ridge regeneration [46]. As this model is not specific to pulp tissues, it should be interpreted as indirect supportive evidence. These results suggest that EMD‐based delivery systems may have potential in dental pulp regeneration. Moreover, the EMD–chitosan complex could achieve sustained release of EMD and inhibit early infection, thereby providing a multifunctional drug delivery system for dental pulp repair, particularly in controlling infection and accelerating tissue repair.

Notably, the core–shell nanofiber membrane encapsulated with EMD, another innovative delivery strategy, can significantly promote the osteogenic differentiation of periodontal ligament stem cells (PDLSCs) through the continuous release of bioactive factors and enhance the mineralization and expression of osteogenic genes (OCN, RUNX2, ALP, and OPN) [47]. Compared with solid nanofibers, the core–shell nanofibers show more durable release characteristics and reduce the initial burst release, thereby improving the therapeutic effect and providing a new drug delivery strategy for RET. As these results are based on periodontal models, their applicability to dental pulp regeneration should be interpreted with caution.

In terms of hydrogel systems, EMD composites also show broad prospects. For example, Attik et al. [48] engineered a synthetic 22‐amino acid starch peptide hydrogel (ADP‐5 hydrogel) that resulted in sustained local release of bioactive factors at potent concentrations, markedly reduced TNF‐α expression, and upregulated levels of anti‐inflammatory factors such as interleukin‐11 (IL‐11). Li et al. [25] used D‐type amino acids to construct an Am‐derived peptide hydrogel (D‐gel), which showed significant biological activity and enhanced odontoblast differentiation and mineralization ability of human DPSCs, with good biocompatibility and stability. Taken together, hydrogel‐based systems may provide a favorable microenvironment, although most available evidence remains preclinical.

Another promising composite system is a hydrogel system made of rhAM, hyaluronic acid (HA), and phytic acid (PA) [49]. This system has excellent mechanical properties, stability, and conductivity, taking full advantage of the proangiogenic properties of Am. The synergistic effect of HA and PA significantly enhanced the biocompatibility and functional properties of the system, allowing the system to adapt to different wound morphologies. This system has significant anti‐inflammatory, pro‐angiogenic, and pro‐cell migration abilities, which provide a new therapeutic strategy for diabetic wound repair. While this system has primarily been evaluated in nonpulp models, its relevance to dental pulp regeneration remains to be established.

Furthermore, leucine‐rich enamel matrix protein peptides (LRAP) have shown great promise in hydrogels. The incorporation of LRAP into the hydrogel not only achieves sustained drug release but also promotes remineralization through its microstructure. LRAP hydrogels have shown clinical potential in reducing cariogenic bacterial infection and promoting enamel repair and regeneration [50], further enhancing the advantages of EMD in combination with biomaterials. Nevertheless, direct evidence supporting pulp regeneration remains limited.

Meanwhile, hydrogels based on Am peptides such as P26, such as the P26‐CS system, have also been shown to promote mineralization [51]. These hydrogels can promote the deposition of calcium and phosphorus crystals and repair enamel and dentin structures, which provides a new idea for the treatment of WSLs and NCCLs. These findings are mainly derived from enamel or dentin repair models. In addition, Hsia et al. [52] have developed a novel light‐responsive rhAm delivery system incorporating HA methacrylate (HAMA) hydrogel to achieve controlled release of rhAm by light, which enhances osteogenic differentiation and tissue repair, providing a novel biomaterial delivery strategy for dental pulp regeneration. However, further validation in pulp‐specific models is required.

In terms of mineralization restoration, El Moshy et al. [53] showed that the combination of nano‐hydroxyapatite (n‐HA) and EMD in agarose hydrogel significantly enhanced mineral deposition during enamel remineralization, especially calcium, phosphorus, and fluoride contents. Therefore, the n‐HA‐EMD hydrogel shows great potential in repairing tooth damage and improving enamel microhardness. The combined effect is better than that of hydrogels using EMD alone, providing a new strategy for pulp regeneration and tooth restoration. However, these findings are derived from enamel remineralization models and should be interpreted as indirect evidence for pulp regeneration.

Using chitosan hydrogels to deliver EMP‐derived peptides, such as P26 and P32, Cai and Moradian‐Oldak [54] found that these complexes could effectively promote dentin mineralization and restore its mechanical properties by closing tubules and promoting collagen mineralization. This study provides a new therapeutic strategy for dental pulp regeneration, and the binding energy of bioactive peptides with the chitosan hydrogel significantly improves dentin mineral density and elastic modulus, contributing to dental pulp restoration. However, the relevance of these findings to pulp regeneration remains indirect.

Furthermore, the application potential of EMD can be further expanded by adding antipollution and antibacterial materials [55]. By combining EMD with materials with anti‐biofilm or immunomodulatory functions, it can effectively maintain the balance of oral microecology, reduce the re‐inflammatory response caused by bacterial infection, and improve the long‐term stability of the regenerated tissue. Nevertheless, the long‐term effects on pulp regeneration require further investigation.

In summary, the EMD‐biomaterial composite strategy appears to provide multiple synergistic effects in structural support, controlled release, and microenvironment optimization, which may enhance the translational potential of EMD in dental pulp regeneration. However, most available evidence remains experimental, and clinical translation in pulp regeneration still requires further validation.

EMD–biomaterial composite systems demonstrate potential advantages in providing structural support, controlled release of bioactive factors, and modulation of the regenerative microenvironment. However, most current evidence is derived from in vitro or nonpulp models, and direct validation in dental pulp regeneration remains limited. Therefore, the translational potential of these systems should be interpreted with caution.

Future studies should further explore the combination of EMD with novel injectable hydrogels, 3D‐printed scaffolds, and intelligent delivery systems to achieve precise and controllable regenerative therapy (Table 1).

**Table 1:** EMD–biomaterial composite systems: classification, functions, and applications.

Composite system	Category	Composition	Functions and advantages	Applications	Research findings	Evidence type	Model
Calcium phosphate/chitosan composite + EMD	Scaffold type	Calcium phosphate, chitosan, recombinant human amelogenin (rhAM)	Enhances EMD release stability, enables sustained release, may inhibit pathogen growth	Oral tissue healing, potential dental pulp repair	Has been reported to support periodontal tissue repair and promote cell proliferation and differentiation	Indirect	Periodontal/in vitro
Calcium silicate materials + EMD	Scaffold type	Calcium silicate materials, EMD	Enhances mineralization and may synergize with the mineralization microenvironment	Potential dental pulp regeneration, dentin mineralization repair	Has been associated with enhanced mineralization and dentin‐like tissue formation	Indirect	Dentin/in vitro
Electrospun functional gradient membrane + DOX + EMD	Drug delivery system	Functional gradient membrane (FGM), doxycycline (DOX), EMD	Enables continuous release of antimicrobial agents, with antibacterial and osteogenic potential	Alveolar ridge regeneration, potential dental pulp repair	Has demonstrated antibacterial and osteogenic potential, may support infection control	Indirect	Bone/in vivo
Core‐shell nanofiber membrane + EMD	Scaffold type	Core‐shell nanofibers, EMD	Provides sustained release of bioactive factors, may enhance osteogenic differentiation and mineralization	Potential dental pulp regeneration, bone repair	Has been reported to improve osteogenic gene expression and therapeutic outcomes	Indirect	Periodontal ligament/in vitro
ADP‐5 hydrogel + EMD	Hydrogel type	22‐amino acid starch peptide hydrogel, EMD	Enables localized sustained release of bioactive factors, may modulate inflammatory responses	Caries repair, enamel repair, regenerative medicine	Has been associated with reduced TNF‐α expression and enhanced anti‐inflammatory activity	Indirect	In vitro
D‐type amelogenin peptide hydrogel + EMD	Hydrogel type	D‐type amino acid‐based amelogenin peptide hydrogel, EMD	May enhance odontogenic differentiation and mineralization, with good biocompatibility	Potential dental pulp regeneration, dentin repair	Has demonstrated biological activity and promotion of mineralization	Direct	DPSCs/in vitro
LRAP hydrogel + EMD	Hydrogel type	Leucine‐rich amelogenin peptide (LRAP), hydrogel, EMD	Enables sustained release, may support remineralization and antibacterial effects	Enamel repair, caries treatment	Has shown potential applicability in enamel repair and remineralization	Indirect	Enamel/in vitro
P26‐CS hydrogel + EMD	Hydrogel type	P26 amelogenin‐derived peptide, chitosan hydrogel, EMD	May promote mineralization and enhance dentin repair properties	White spot lesion (WSL), NCCL repair	Has been associated with increased mineral deposition and improved mechanical properties	Indirect	Enamel/dentin
rhAM + HA + PA hydrogel + EMD	Hydrogel type	Recombinant amelogenin (rhAM), hyaluronic acid (HA), phytic acid (PA), EMD	May promote angiogenesis, anti‐inflammatory effects, and cell migration	Diabetic wound healing, potential dental pulp regeneration	Has demonstrated anti‐inflammatory and pro‐angiogenic potential	Indirect	Soft tissue/in vivo
n‐HA‐EMD hydrogel	Hydrogel type	Nano‐hydroxyapatite (n‐HA), EMD, agarose hydrogel	May enhance mineral deposition during remineralization	Tooth repair, enamel microhardness enhancement	Has been associated with increased mineral content and improved enamel properties	Indirect	Enamel
P26 and P32 peptide hydrogel + EMD	Hydrogel type	P26/P32 peptides, chitosan hydrogel, EMD	May promote dentin mineralization and restore mechanical properties	Potential dental pulp regeneration, dentin repair	Has been reported to improve mineral density and elastic modulus	Indirect	Dentin
EMD with antibacterial/antifouling materials	Other type	EMD combined with antibacterial or antifouling materials	May help maintain oral microecological balance and reduce inflammation	Dental pulp repair, tooth repair	Has been associated with improved long‐term stability of regenerated tissues	Indirect	In vitro/in vivo

*Note:* EMD, Emdogain, recombinant amelogenin (rhAM), and amelogenin‐derived peptides represent related but not equivalent biomolecular interventions, and their biological effects should be interpreted accordingly. Evidence type is categorized as direct (pulp‐related models) or indirect (nonpulp models).

## 6. Clinical Applications and Translational Research

Before discussing clinical translation, it is important to critically appraise the current evidence base. Existing studies exhibit substantial heterogeneity in the EMD composition, concentration, delivery systems, and experimental models. Moreover, many studies are limited by small sample sizes, a lack of standardized outcome measures, and potential sources of bias, including insufficient blinding and publication bias. Furthermore, the proprietary nature of EMD and variability in experimental protocols may affect reproducibility across studies.

Although most research on EMD in regenerative pulp therapy (RET) remains at the experimental and animal study stages, preclinical studies have demonstrated the potential for clinical application. Matsumoto et al. [56] evaluated the effect of Emdogain gel in repairing periapical defects using a rat model and found that EMD significantly promoted tissue repair in the periapical region, enhancing new cementum formation. Alhazzazi et al. [57] reported that applying Am in combination with platelet‐rich plasma (PRP) to young permanent teeth with periapical periodontitis significantly promoted the regeneration of periodontal and pulp tissues. Observations at 1 and 3 months postsurgery revealed apical foramen closure, cementum and alveolar bone deposition, and the formation of vascularized pulp‐like tissue [57]. Mounir et al. [37] further demonstrated that EMD‐treated root canals exhibited dense regenerative tissue in the apical region, forming a neovascularization network in the root canal and pulp chamber, accompanied by the regeneration of nerve and sensory structures. These findings provide preliminary histological and experimental support for the potential role of EMD in pulp repair; however, most evidence remains derived from animal models or limited clinical observations.

Importantly, these outcomes should be interpreted with caution as histological evidence from regenerative endodontic studies indicates that the newly formed tissues are often pulp‐like, fibrous, or mineralized in nature, rather than fully recapitulating the native pulp–dentin complex [2]. True pulp regeneration is generally defined by the restoration of vascularized and innervated pulp tissue with odontoblast‐like cell alignment and dentin formation, whereas reparative outcomes typically involve apical closure, mineralized barrier formation, or fibrous tissue ingrowth without full functional recovery. This distinction is particularly relevant when interpreting EMD‐related findings because apical closure, mineralized tissue deposition, or tissue ingrowth do not necessarily indicate restoration of native pulp architecture and function.

Despite the promising results from animal experiments, several challenges remain for the clinical translation of EMD. First, the precise molecular mechanisms and signaling networks underlying EMD’s effects have not been fully elucidated, and interindividual differences in biological responses may result in inconsistent efficacy. Second, systematic studies on the interaction of EMD with conventional endodontic materials, such as root canal disinfectants, filling materials, and carrier scaffolds, are still lacking. Additionally, the sample sizes in current clinical studies are limited, and there is a lack of long‐term follow‐up and randomized controlled trial data. These limitations highlight the gap between experimental findings and clinical implementation.

EMD should also be interpreted within the broader landscape of regenerative strategies. Compared with PRP/PRF‐based approaches, EMD may offer a more standardized biologic formulation, whereas platelet concentrates have the practical advantage of autologous origin and are already more familiar in clinical RET protocols. In contrast, cell‐based therapies may provide a higher degree of control over the cellular component of regeneration, but they are associated with greater complexity in cell sourcing, expansion, delivery, and regulation. At present, the available evidence is insufficient to conclude that EMD is superior to PRP/PRF or cell‐based strategies; rather, EMD may be better viewed as one potentially useful biologic component within a broader regenerative framework.

Further translational issues should also be acknowledged. Notably, potential immunologic issues should be considered. As EMD is derived from porcine EMPs, its xenogeneic origin may raise concerns regarding immunogenicity, particularly when applied within the root canal system. Although EMD has demonstrated a favorable safety profile in periodontal applications, its immunologic behavior in pulp regeneration, including the potential for antibody formation or altered host responses, remains insufficiently characterized. Because commercially used EMD is derived from porcine EMPs, regulatory classification, batch consistency, and product standardization remain relevant considerations for future clinical use. In addition, cost‐effectiveness has not yet been adequately evaluated, and this may become an important determinant of whether EMD‐based approaches can be integrated into routine clinical practice.

Although EMD shows promising biological and preclinical effects in regenerative endodontics, its clinical translation remains limited by insufficient high‐quality evidence, heterogeneity of study designs, and unresolved regulatory and immunologic concerns. Further, well‐designed clinical studies are required to establish its safety, efficacy, and comparative advantages.

## 7. Summary and Future Directions

EMD has been reported to exhibit a range of biological effects relevant to regenerative dental pulp therapy, including the promotion of stem cell migration and differentiation, support of angiogenesis and neurogenesis, and regulation of the regenerative microenvironment through immunomodulatory mechanisms.

Despite these promising effects, the clinical translation of EMD faces several challenges. These include standardization issues due to its animal origin, the unclear long‐term efficacy and dose–response relationship, and variations in its effects under different pathological conditions. Moreover, most current evidence remains preclinical, and the extent to which these effects can be translated into predictable clinical outcomes remains uncertain.

Future research should focus on the following key areas:

1. In‐depth exploration of molecular mechanisms: further studies are needed to clarify the precise molecular mechanisms of EMD and its interactions with cellular signaling pathways. While existing evidence indicates that EMD acts through multiple pathways, the relative contribution and interaction of these pathways in pulp regeneration remain to be fully elucidated.
2. Optimization of delivery and controlled release systems: developing novel delivery systems to enhance the stability and bioavailability of EMD is crucial. Integrating EMD with biomaterials, such as injectable hydrogels and 3D‐printed scaffolds, could provide precise control over growth factor release, optimizing the local microenvironment and enhancing the regeneration process. However, these strategies require further validation in clinically relevant models.
3. Rigorous clinical trials: multicenter, randomized controlled clinical trials are essential to systematically assess the long‐term efficacy, safety, and dose–response relationship of EMD. These trials should involve large sample sizes, long‐term follow‐up, and robust validation to ensure the successful translation of laboratory findings into clinical practice.
4. Promoting interdisciplinary collaboration: strengthening collaboration between biomaterials engineering, immunology, and clinical medicine is vital for overcoming the challenges in the clinical application of EMD. Such collaboration may facilitate the translation of experimental findings into clinical practice.
5. Clarifying regenerative endpoints and comparative value: Future studies should more clearly distinguish true pulp regeneration from reparative healing and should directly compare EMD‐based approaches with PRP/PRF, cell‐based therapies, and other regenerative strategies. Such comparisons will be important for defining the specific clinical value of EMD rather than assuming broad superiority.
6. Addressing translational feasibility: Future work should also evaluate regulatory requirements, product standardization, and cost‐effectiveness as these factors will influence whether EMD can move beyond experimental promise and become clinically practical.

With advancements in biomaterials technology and regenerative medicine, EMD may represent a promising strategy for dental pulp regeneration; however, its clinical effectiveness and long‐term outcomes require further validation. Continued interdisciplinary research and technological development are expected to support the optimization of EMD‐based regenerative approaches.

## Funding

This study was supported by the Dean’s Research Fund of Jinan Stomatological Hospital (2019‐02).

## Conflicts of Interest

The authors declare no conflicts of interest.

## Data Availability

This manuscript presents a narrative review of the current evidence on enamel matrix derivatives (EMD) in dental pulp regeneration. No new data were generated or analyzed in this study. All data discussed are derived from previously published studies, which are appropriately cited in the reference list. Where applicable, both direct and indirect evidence are considered to provide a comprehensive overview.
